# Accurate disaster entity recognition based on contextual embeddings in self-attentive BiLSTM-CRF

**DOI:** 10.1371/journal.pone.0318262

**Published:** 2025-03-26

**Authors:** Noor E. Hafsa, Hadeel Mohammed Alzoubi, Atikah Saeed Almutlq

**Affiliations:** Department of Computer Science, College of Computer Science and Information Technology, King Faisal University, Al Ahsa, Saudi Arabia; Dalian Maritime University, CHINA

## Abstract

Automated extraction of disaster-related named entities is crucial for gathering pertinent information during natural or human-made crises. Timely and reliable data is vital for effective disaster management, benefiting humanitarian response authorities, law enforcement agencies, and other concerned organizations. Online news media plays a pivotal role in disseminating crisis-related information during emergencies and facilitating post-hazard disaster response operations. To extract relevant named entities, contextual embedding features prove instrumental. In this study, we investigate the automatic extraction of disaster-related named entities from an annotated dataset of 1000 online news articles. These articles are carefully annotated with 14 crisis-specific entities obtained from relevant ontologies. To generate contextual vector representations of words, we construct a novel word embedding model inspired by Word2vec. These contextual word embedding features, combined with lexicon features, are encoded using a novel contextualized deep Bi-directional LSTM network augmented with self-attention and conditional random field (CRF) layers. We compare the performance of our proposed model with existing word embedding approaches. Through extensive evaluation on an independent test set of 200 articles that includes more than 80,000 tokens, our context-sensitive optimized NER model achieves impressive results at the sentence level. With a Precision of 92%, Recall of 91%, Accuracy of 87%, and an F1-score of 92%, our model outperforms those utilizing general and non-contextual word embeddings, including fine-tuned and contextual BERT models, showcasing its superior performance.

## Introduction

Named Entity Recognition (NER) is a vital task that involves identifying named entities in text across predefined semantic categories, including person, location, organization, and others [1]. NER serves not only as an information extraction tool but also finds utility in various natural language processing (NLP) applications, such as question answering, information retrieval, co-reference resolution, text understanding, automatic text summarization, machine translation, and topic modeling [2]. Given the substantial volume of structured and unstructured textual content generated daily by news providers and companies publishing online news articles, effectively leveraging tools and techniques for text search and indexing becomes crucial. Moreover, the rapid flow of data, contextual comprehension, time sensitivity, scalability limitations, as well as the emotional and psychological impact, present additional obstacles to the manual processing of news data. Therefore, the integration of automated tools and technologies, including natural language processing, machine learning, and AI-driven algorithms, becomes imperative for facilitating the automatic extraction of information during disaster events. By applying automated NER to news articles, we can accurately identify the named entities contained within them. This enables prompt detection of the required information with a high level of reliability and trustworthiness [3].

In recent years, the world has experienced an increase in natural and man-made disasters, posing challenges for manually processing the vast amount of data available from news and social media during these events. Effective disaster management relies on humanitarian response organizations and other concerned entities collecting reliable disaster-related information as a primary step. Both social and online news media may prove invaluable during disaster events, serving multiple purposes such as real-time information sharing, the rapid dissemination of emergency alerts and warnings, crowd-sourcing and crowdsensing, facilitating communication and coordination, promoting public awareness and education, fostering emotional support and community building, and aiding situational awareness. Additionally, online news media provides reporting on relief efforts, eyewitness accounts and testimonials, and conducts investigative journalism to monitor relief organizations’ efforts, and analyze the disaster impact and long-term implications. Significantly, during disaster events, news media information holds greater reliability owing to its adherence to editorial standards, meticulous source verification, news accountability, the credibility of reporters, editorial gatekeeping, and the presence of a verification feedback loop. Extracting named entities related to disasters and crises from news articles can therefore greatly assist in managing post-disaster activities. Exploiting cutting-edge technology, automated extraction of information from online news media texts proves to be more effective than laborious manual extraction. This automated information extraction involves identifying named entities such as individuals, organizations, and locations in the source texts. In the context of crisis and disaster management, relevant named entities can be extracted from disaster-related ontologies. Examples include hazard types, locations, dates, death tolls, affected populations, infrastructure damages, and other pertinent information.

In summary, this research encompasses the following key contributions:

1. Generation of a meticulously annotated NER dataset specifically crafted for disaster management, which, to the best of our knowledge, is the first of its kind. This dataset consisting of disaster-related news articles, is created using vocabulary specifications extracted from disaster-oriented ontologies, ensuring the identified entities are directly relevant to disaster scenarios.
2. Construction of a novel Word2Vec-inspired contextual word embedding model, tailored specifically for the context of disaster events. This model generates contextual vector representations of word tokens capturing the semantic relationships between words in the disaster domain.
3. Development of a custom character embedding model utilizing Convolutional Neural Networks (CNN). This model extracts character-level features, enabling the recognition of patterns and structures within words, further enhancing the accuracy of named entity recognition.
4. Creation of an optimized self-attention based Bi-LSTM CRF model (BiLSTM-ATTN-CRF). This model integrates the context-specific word embedding and character embedding features with other relevant NLP features, resulting in a powerful and comprehensive framework for recognizing disaster-related named entities.
5. Conducting comparative analyses to evaluate the performance of the BiLSTM-ATTN-CRF model and investigate the effectiveness of contextual word embeddings. The latter analysis focuses on comparing the utilization of context-specific word embedding features versus general word embedding features in the task of recognizing disaster-related named entities.

The remainder of this document is structured as follows: The Related Works section offers a concise overview of relevant literature in the field. The Methods section provides a detailed explanation of the dataset preparation and the methodology employed. In the Results section, we present the findings obtained from our research. A comprehensive analysis and interpretation of these findings are provided in the Discussion section. Finally, we conclude by summarizing the key findings and discussing the potential impact of our research.

## Related works

Over the past few years, the widespread adoption of deep learning in natural language processing (NLP) has become increasingly common. Recurrent Neural Networks (RNN) and other advanced models are now frequently employed to address various tasks such as text classification, named entity extraction, and language modeling. Shen et al. [4] explored the combination of deep learning and active learning to reduce the reliance on labeled training data. They introduced active learning as an alternative to random labeling, allowing for the selection of specific samples to be labeled. Additionally, they proposed a lightweight deep neural network architecture, the CNN-CNN-LSTM model, for the Named Entity Recognition (NER) task. This model stands as one of the state-of-the-art performers, demonstrating high efficiency in terms of computation and exceptional performance on standard datasets. Their research focused on the CoNLL-2003 English and OntoNotes-5.0 English datasets, achieving outstanding results with a 99% F1 score evaluation metric.

Lample et al. [5] addressed the challenge of limited supervised training data for generalizing NER models across multiple languages. They presented two neural architectures, one based on the bi-directional LSTM-CRF model and the other on the transition stack LSTM model, which did not rely on language-specific resources or features. The models were tested on four different languages—English, Dutch, German, and Spanish—demonstrating their ability to generalize across diverse languages. The results on the CoNLL-2002 and CoNLL-2003 datasets showcased state-of-the-art performances for both models. The LSTM-CRF model achieved F1-measure scores of 90.94 (English), 78.76 (German), 81.74 (Dutch), and 85.75 (Spanish), while the S-LSTM model achieved F1-measure scores of 90.33 (English), 75.66 (German), 79.88 (Dutch), and 83.93 (Spanish). Al-Smadi et al. [6] focused on named entity recognition in the Arabic language, which poses challenges due to its complex nature. They employed transfer learning using a deep neural network, constructing a Pooled-GRU Model combined with a Multilingual Universal Sentence Encoder to extract Arabic named entities from text. The proposed model was compared to the baseline Bi-LSTM-CRF model from their previous research, aiming to achieve enhanced results. The evaluation was performed using the WikiFANEGold dataset, consisting of news-based and Wikipedia-based fine-grained Arabic named entity gold corpus. The proposed model achieved an F1-measure of 90.25% and an accuracy of 91.20%, showcasing a significant improvement of approximately 17% compared to their previous work.

Cruz et al. [7] focused on online news media as a valuable source of information. They created a dataset using the Pilipino Star NGAYON newspaper, extracting news articles related to natural disasters in the Philippines, particularly in 2014 when the country experienced major natural disasters. The collected data, written in Filipino, underwent cleaning and filtering using Notepad++ to align with the ConLL-2003 format commonly utilized by NER tools. Deep learning techniques were implemented using Tensor Flow, an open-source software library, to perform named entity recognition. The model achieved impressive results, with an accuracy of 99.65% and an F1-measure of 75.71%.

Another study by Domala et al. [8] aimed to dynamically identify disaster-relevant news from English news websites using natural language processing techniques and machine learning. Their system automatically scraped news from various sources, including Times of India, NDTV India, and Indian Express, at five-minute intervals. The scraped data formed the training dataset for classification purposes. Machine learning classifiers such as Naive Bayes, logistic regression, SVM, Extreme gradient boosting, and random forest were employed to classify news into relevant or irrelevant categories. The highest precision, recall, and F1 scores were achieved by logistic regression and SVM algorithms. Ultimately, logistic regression was chosen as the preferred algorithm due to its lower false positive rate compared to SVM, resulting in the best overall performance.

In a study by Jansson et al. [9], the researchers aimed to address the challenge of identifying named entities in noisy text, such as user tweets, online reviews, and social media posts that contain unusual or previously unseen content. They proposed an approach that combines LDA topic modeling with deep learning at both the word and character level embeddings. The LDA topic modeling technique was used to classify each tweet or post into a specific topic, and this topic classification was utilized as a feature for each word in the text. The experimental results demonstrated a performance of 41.81 in terms of F1-measure for identifying entities and 40.57 for surface forms. In another study by Wei et al. [10], the authors focused on the challenges of identifying entity names in biomedical text, which often includes a wide range of phrases and abbreviations related to drugs, diseases, genes, chemicals, and proteins. They utilized the JNLPBA corpus dataset, which encompasses five entity types: DNA, RNA, Cell Type, Cell Line, and Protein. Their model achieved an impressive F1-score of 73.50 on the JNLPBA dataset.

Two recent studies have concentrated on named entity recognition (NER) within the context of local language modeling. The first study, conducted by Sornlertlamvanich et al. [11], centered on Thai NER and employed a BiLSTM-CNN-CRF model enhanced with a Thai character cluster (TCC) module integrated into the character representation layer of the Bi-LSTM network. To enhance the accuracy of the NER model, they employed iterative training, progressively improving named entity annotation by reducing errors at the word segmentation level. Through this iterative training process, the model achieved an impressive *F*_1_-score of 89.22%. In the second study, Zhao et al. [12] focused on leveraging the unique characteristics of Chinese characters to extract agricultural named entities. They aimed to augment the semantic information of Chinese characters by extracting corresponding radical and stroke features, resulting in a more comprehensive vector representation of the target words and enhancing NER precision. By utilizing these enriched feature sets, a BiLSTM-CRF model achieved an outstanding *F*_1_-score of 95.56%.

However, our extensive literature survey revealed two significant research gaps. Firstly, the existing research primarily concentrates on utilizing social media data as unstructured text for extracting named entities in the context of disaster management. While online news articles are considered reliable sources of information, there is a dearth of research specifically addressing this aspect. Secondly, the analysis of the impact of context-specific embedding features on the performance of named entity recognition tasks is largely absent in the current literature.

To address these gaps, our research aims to consider online news articles as semi-structured texts for extracting disaster-related named entities. We annotate a novel dataset of 1000 disaster-related news articles with 14 different crisis-relevant entities using vocabulary specifications extracted from MOAC (Management Of A Crisis) [13] and Empathy [14] ontologies. Additionally, we construct a context-specific word embedding model based on the Word2vec [15] algorithm using a collection of CNN (https://edition.cnn.com//) news articles on disaster events. Subsequently, we develop a novel Bi-LSTM network with self-attention and CRF layers, incorporating contextual word embedding, character embedding, and other NLP features. We analyze the effectiveness of contextual word embedding features in extracting disaster-specific named entities compared to general embedding features.

## Materials and methods

### Dataset preparation

**Fig 1:**
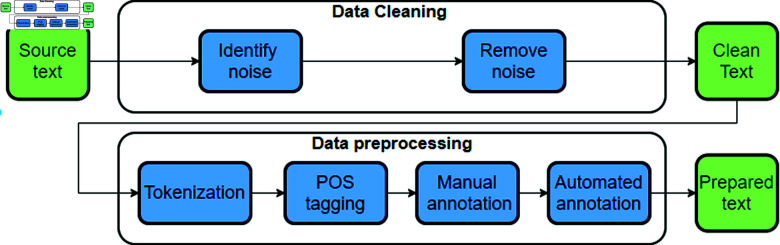
The news articles dataset pre-processing steps are illustrated.

In this study, we utilize a comprehensive dataset of news articles focused on natural disasters, covering an eight-month period. The dataset, consisting of 34,000 articles in English, was compiled using AYLIEN’s News API (https://aylien.com/product/news-api). The articles span from May 2019 to December 2019. To facilitate effective natural language processing (NLP) modeling, we carefully prepare the dataset, as detailed in Fig 1, outlining the specific steps involved in the dataset preparation process.

#### Noise removal.

The online articles dataset undergoes a series of preprocessing steps to facilitate subsequent NLP modeling. These steps encompass removing blank or unnecessary rows, identifying and eliminating duplicated articles, and ensuring optimal data processing by removing extra spaces.

#### Word tokenization.

As part of the data preparation stage, one of the initial steps involves word tokenization of the training sentences, including the punctuation marks. In this stage, the text is divided into individual words or tokens. This step lays down the foundation for further text processing tasks such as parsing, POS tagging, and others. By segmenting the text into tokens, the NLP model can better understand the linguistic content of the input data.

### Dataset annotation

The dataset annotation occupies a significant part in the Dataset preparation step in the NER study at hand, considering that the initial dataset collected lacked annotations. To annotate our dataset at the word level, we leverage disaster-related vocabularies extracted from relevant ontology databases. This annotation process involves classifying each word in every sentence of the article dataset, thereby providing the named entities required for the NER task in our study. The annotation procedure follows a semi-supervised approach, divided into two steps. Initially, we create a gold standard corpus consisting of manually annotated articles. The manual annotation process entails various stages, including reading the articles, comprehending the classes and properties within the ontology, identifying entities suitable for annotation, mapping these entities to appropriate classes, and finally subjecting the annotation to expert review for validation. In the second stage, we employ AI-inspired annotation tools to automatically extract disaster-related named entities from the articles. However, the automated annotation is not considered final and undergoes an additional review by domain experts to ensure the accuracy and reliability of the annotated entities. This iterative process of human-in-the-loop verification strengthens the quality of the annotation results.

#### Gold standard corpus by manual annotation.

In this particular step, we performed manual annotation on a subset of articles from the original dataset. The annotation process involved utilizing disaster-related vocabulary specifications extracted from MOAC [16] and Empathi [14] ontologies. These ontologies encompass various classes and properties relevant to disasters, which were analyzed and employed to assign appropriate disaster classes to the words within the articles comprising our gold standard corpus. For instance, consider the word ‘Earthquake’, which was mapped to the ‘NaturalDisaster’ class as specified in the MOAC ontology. A total of 50 articles were meticulously annotated at the word level to create the gold standard corpus. To ensure the reliability of the manual annotation, a domain expert thoroughly reviewed the process, encompassing two key tasks: 1) verifying the correctness of the word-level mapping to disaster classes, 2) modifying the mapping if a more suitable class was identified, and so on. The rigorous review and validation conducted by the domain expert guarantee the accuracy and quality of the manual annotation. The expertise of the domain expert ensures that the assigned disaster classes align appropriately with the context and content of the articles, refining the overall reliability of the annotation process.

#### Automated extraction of named entities.

The extraction of named entities from the disaster article dataset, consisting of 950 articles, was conducted through an automated process. The gold standard corpus, described earlier, served as the reference dataset for this task. Several automated annotation tools were identified, including UBIAI [17] and Tagtog [18], which employ machine learning algorithms to learn from pre-annotated texts and classify named entities in unannotated texts. After careful consideration, the Tagtog tool was chosen for the automated annotation task. This decision was based on its built-in machine learning module, which facilitated the learning of entity class mapping from the reference dataset. The Tagtog tool provided a convenient and effective solution for automating the annotation process, ensuring accurate identification and classification of named entities in the unannotated texts. By leveraging the capabilities of the Tagtog tool and its machine learning module, the automated extraction of named entities from the disaster article dataset was efficiently performed, aligning with the standards set by the gold standard corpus.

**Table 1:** A summary of Disaster-related Entity class names including a brief description and the source ontology is listed.

Entity	Description	Ontology
**NaturalHazards**	A term represents a threat of a naturally occurring event that will have a negative effect on people or the environment.	MOAC
**Location**	A certain location “where" the hazard takes place.	Empathi
**Date**	Indicates a particular date	MOAC
**Floods**	An overflow of an expanse of water that submerges land.	MOAC
**Fire**	An uncontrolled burning which has the potential to cause physical damage on human life, health, property or ecology.	MOAC
**Death_and_Toll**	The number of people died in the event of natural and man-made disasters.	Empathi
**InfrastructureDamage**	Refers to the damage of physically existing basic facilities, services, and installations needed for the functioning of a community or society.	MOAC
**CollapsedStructure**	A term that defines a totally damaged state of a structure resulting from human or natural phenomenon.	MOAC
**RoadBlocked **	Indicates a road that is blocked by a barricade caused by natural disaster or set up by human.	MOAC
**MissingPersons**	The persons whose whereabouts are unknown after sudden onset of disaster.	MOAC
**PowerOutage **	A term which refers to electrical power failure, which means a short or long-term loss of the electric power to an area.	MOAC
**AffectedPopulation**	Indicates the communities and peoples affected by the occurrence of natural or man-made disasters.	Empathi
**WaterShortage**	A term used to refer to the situations during which available water resources are insufficient to meet human demand.	MOAC

### Disaster class description

The annotation process described earlier successfully extracted a total of 32,747 entries representing 13 different disaster classes from the news article dataset. Table 1 provides an overview of the Disaster entity class names along with their corresponding descriptions.

**Fig 2:**
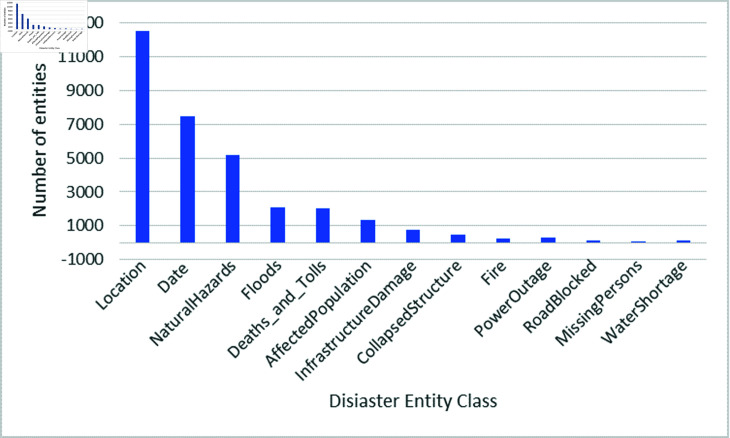
A bar plot showing the number of 14 Disaster-related entity types (in hundreds) in the news articles dataset.

**Fig 3:**
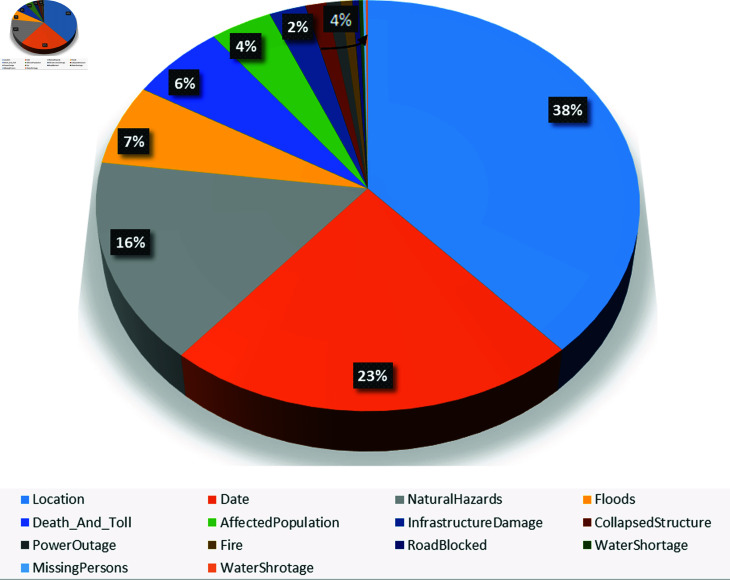
A pie diagram illustrating the percentage distribution of 14 Disaster-related entity types in the news articles dataset.

Additionally, Figs 2 and 3 present a bar and a pie chart, respectively, illustrating the distribution of annotated entities across the Disaster classes. The figure highlights that the majority of the named entities identified in the articles belong to the following classes: ‘NaturalHazard’, ‘Location’, ‘Date’, ‘Floods’, ‘Death_And_Toll’, and ‘AffectedPopulation’. These classes accounted for a significant portion of the annotated entities. Conversely, the classes with lower occurrences were ‘InfrastructureDamage’, ‘CollapsedStructure’, ‘Fire’, and ‘PowerOutage’. Furthermore, the ‘RoadBlocked’, ‘MissingPersons’, and ‘WaterShortage’ entity classes had the lowest frequencies among all the annotated entities. The distribution of entities across the Disaster classes provides valuable insights into the prevalence of different types of disasters and their associated entities within the news article dataset. It helps identify the focus areas and the relative importance of each Disaster class in the context of the dataset.

### Comprehensive word embedding features

#### Syntactic-level embedding.

Grammatical features, such as Parts of Speech (POS) tagging and Casing information, are incorporated into the NER modeling to improve its performance. POS tagging offers valuable insights into the proper position of words within a sentence, which significantly influences the sentence’s meaning. Subsequently, a POS tagging process is applied to the tokenized words, assigning each word in the sentence to its respective part of speech category, such as noun, verb, adverb, etc. To accomplish these tasks, we utilized the NLTK Python library. Table 2 provides an illustrative example of word tokenization and POS tagging applied to a sentence from the article dataset. This enables us to extract crucial information about the syntactic role of each word, aiding in accurate classification.

Additionally, word-specific casing information is captured by classifying each word in a sentence in ‘numeric’, ‘mainly_numeric’ (fraction of digits  >  50%), ‘allLower’, ‘allUpper’, ‘initialUpper’, and ‘contains_digit’ classes through a lookup function. This casing information plays a vital role in the NER task by identifying the presence of proper nouns within the input texts. By considering the actual casing (capitalization) of words, we can effectively pinpoint the locations of proper nouns, often indicative of named entities. Including this syntactic feature further enhances the accuracy of named entity classification providing valuable cues for identifying and categorizing entities. By exploiting these grammatical features, such as POS tagging and casing information, our NER model gains a deeper understanding of the linguistic structure of the text. This, in turn, enhances the accuracy and effectiveness of the named entity classification process.

#### Word-level embedding.

In order to extract word-level embedding features for each query word, we develop a tailored word embedding model specifically designed for the context of disasters. Our objective is to acquire a contextual representation of each query word that encompasses both syntax and semantic information. To achieve this, we employ word2vec [15], a widely utilized word embedding algorithm. Word2vec utilizes a simple neural network model that can learn word embedding features from extensive collections of contextual texts. In the following sections, we provide an overview of the steps involved in constructing our customized word embedding model.

**A. Dataset collection.** For the word embedding model dataset, we collected a comprehensive set of 9,800 news articles from the CNN (https://edition.cnn.com//) news website. These articles focus on disasters and encompass the timeframe from 2010 to 2021. Our dataset includes a diverse range of natural disasters, such as floods, tornadoes, earthquakes, and more, occurring in various locations worldwide during the specified period. To extract the articles from the CNN website, we utilized the Octoparse web scraping tool [19]. By leveraging this tool, we were able to efficiently gather the necessary data for our word embedding model.

**Fig 4:**
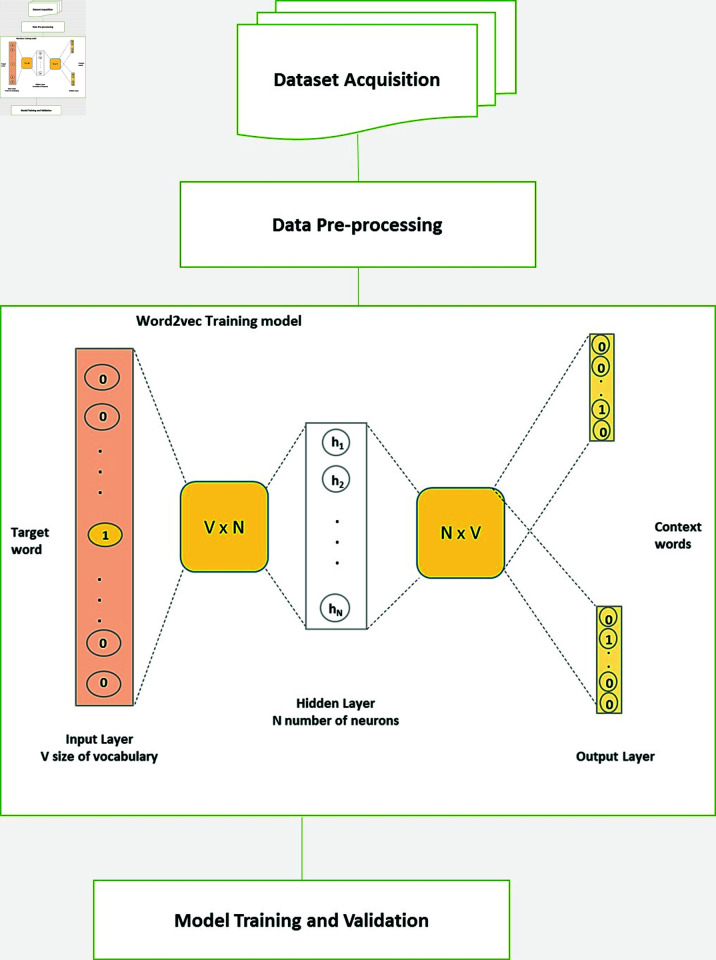
The word embdedding model development process explained. | *V* |  denotes the cardinal of the vocabulary (i.e. the different words in the corpus) and N denotes the dimension of the embedding vectors (i.e. the hidden layer has N dimensions) where C represents the context window’s size.

**B. Embedding model Construction.** The major steps in building the custom word embedding model are presented as a flow chart in Fig 4. The steps are summarized in the subsequent paragraphs below.

**Table 2:** An example word tokenization and POS tagging of a training sentence.

Token	POS tagging	
‘The’	DT	Determiner
‘intensity’	NN	Noun, singular
‘of’	IN	Preposition
‘the’	DT	Determiner
‘drought’	NN	Noun, singular
‘in’	IN	Preposition
‘New’	NNP	Proper noun, singular
‘South’	NNP	Proper noun, singular
‘Wales’	NNP	Proper noun, singular
‘was’	VBD	Verb, past tense
‘left’	VBN	Verb, past participle
‘soil’	NN	Noun, singular
‘moisture’	NN	Noun, singular
‘levels’	NNS	Noun, plural
‘severely’	RB	Adverb
‘depleted’	VBD	Verb, past tense
‘.’	.	.

*Dataset preparation.* To prepare the dataset for training the word embedding model, we conducted essential data pre-processing steps such as tokenization and noise removal, followed by transforming the article texts into a word vector representation. Additionally, we converted numeric values into their string forms, for instance, transforming the number ‘300’ into the word ‘Three Zero Zero’. The resulting word vectors from these procedures were consolidated into a single matrix serving as the training data for the embedding model. In total, the training matrix encompassed six million words extracted from 9800 articles, ensuring a comprehensive and representative dataset for the word embedding model’s training process.

**Fig 5:**
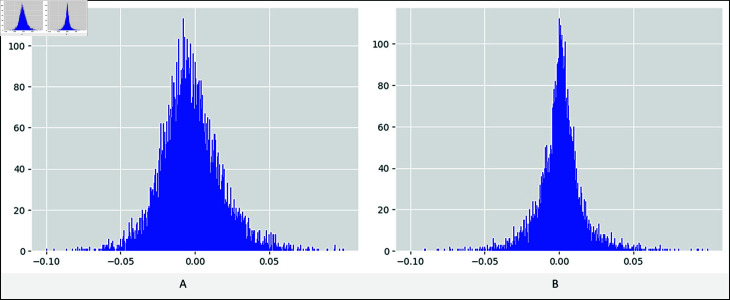
The histograms of the word embedding vector distributions are illustrated. A. Contextual embedding B. Word2vec embedding.

*Model training and validation.* We trained our custom word embedding model using the word2vec model architecture [15]. Specifically, we employed the continuous skip-gram technique to generate numeric vector representations for the words in the training texts. To optimize the performance of the embedding model on the disaster-related training words, we fine-tuned the hyperparameters, focusing on the ‘embedding size’ and ‘learning rate’ variables. During training, the model aimed to learn the similarity between word vector representations, utilizing cosine distance to measure proximity among the words. After careful tuning, we determined the optimal values for the ‘embedding size’ and ‘learning rate’ parameters as 100 and 0.15, respectively. To evaluate the efficacy of our custom word embedding model, we selected a development set comprising 100 articles and applied the Pearson’s correlation coefficient (PCC) evaluation metric, recommended in [20]. The research highlighted the utility of PCC in measuring the similarity between two embedding representations when their mean vector distributions are centered at zero. Through a histogram analysis comparing the mean distributions of initial word representations and those of the resultant embedding representations by our custom word embedding model, we identified consistent trends (as depicted in Fig 5). Subsequently, we utilized the PCC metric to assess the similarity between each word, represented as a numeric vector in the annotated vocabulary, and the corresponding numeric row (representing a word) in the resulting word embedding matrix. Notably, the mean PCC value in this evaluation study peaked at 60%.

In the NER model training sentences, each query token is assigned a numeric value by the word embedding model, reflecting its proximity to similar contextual words. This numeric value serves as the word embedding feature within the NER model, capturing the contextual representation of the query word.

#### Character-level embedding.

In context-oriented structured text analysis, sub-word or character-level information assumes a crucial role. This is particularly evident in disaster articles, where unique abbreviations and terminologies are often used to mention entities that are out-of-vocabulary (OOV) and appear with lower frequency. While word embedding provides valuable numeric vector representations for words within the context, it may struggle to generate meaningful features for OOV words. In contrast, character-level representations of each word excel at extracting orthographic and morphological information from unconventional words. Consequently, incorporating character encoding information becomes essential for creating a comprehensive word representation for each query word. By utilizing character-level information, we enhance the overall effectiveness of the word representation in capturing the intricacies of the text.

**Fig 6:**
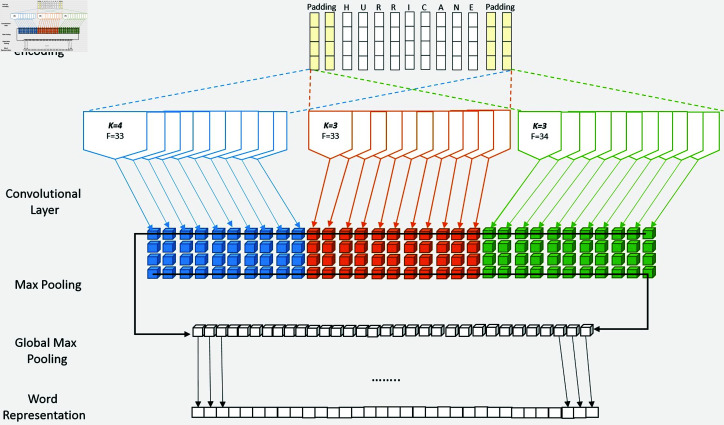
The illustration of the character embedding model.

We utilized a CNN-based neural network to generate the character embedding model, drawn from the work of Ma et al. [21]. The character-level embedding of each word token involves several steps. First, the characters are ordinal or integer encoded, resulting in a one-dimensional vector of 52 elements. We set the character vector at a moderate size of 52 to lessen computational efforts while retaining substantial informativeness. The character encoding is performed by mapping the characters to an alphabet that includes upper and lower-case letters, digits, and special characters (i.e. 0123456789abcdefghijklmnopqrstuvwxyzABCDEFGHIJKLMNOPQRSTUVWXYZ./,-_()[]{}!?:;#’\"\%$‘&=*+@ ∧ ∼ “”*μ*\n©£°\xad¢’ƒ | *′*–). Next, the encoded character vectors undergo a series of operations. They are subjected to three parallel 1D-convolution layers, utilizing a set of kernels [4, 3, 3] and filters [33, 33, 34]. Notably, the optimal kernel and filter sizes in this approach are meticulously chosen through trial-and-error methods. Following the convolutions, max-pooling, and global max-pooling layers are applied to reduce the dimensionality of the convoluted character vectors. As a result of these processes, each word token is represented by a 100-dimensional numeric vector obtained from the character embedding. The character-level embedding model is illustrated in Fig 6.

### Bi-LSTM augmented with self-attention and CRF layers

In the current problem setting, our objective is to assign the appropriate disaster class to each word token within a sentence. This task can be classified as a sequence labeling problem that necessitates exploiting both past and future contextual information. Historically, LSTM-based recurrent neural networks have demonstrated exceptional effectiveness in addressing such sequence labeling challenges, particularly in named entity extraction tasks [22–24]. Specifically, Bi-LSTM networks have the ability to learn from sequence data by utilizing hidden neurons to capture both past and future contextual information. These historical and future context details serve as valuable features for predicting the label of the current word token. Additionally, a self-attention recurrent neural network can effectively capture the long-range dependencies among words within a sentence. In the case of sequence tagging problems, incorporating self-attention at the top layer of the model enables it to learn the implicit dependency information between different tags [25]. For instance, consider the training sentence: “In neighboring Guangdong province (location), seven people were killed (Death_and_toll) and one was missing (MissingPersons) as heavy rain (NaturalHazards) destroyed roads (InfrastructureDamage) and toppled houses (CollapsedStructure)." In this example, the “NaturalHazard" entity exhibits internal relationships with all the other disasterrelated entity tags. By using a Multihead SelfAttention layer, the model can effectively learn the distant relationships among different entities and capture the internal dependencies between them. This is achieved by employing a special attention situation where Query=Key=Value. This specialized attention mechanism is applied to each word in each sequence and across all sequences, enabling the model to learn important relationships and dependencies [26]. The refined representation of the input vectors from the self-attention layer then undergoes the CRF layer, which utilizes the ‘Viterbi’ algorithm [27] for probabilistic inference of the entity labels. As a probabilistic graphical model, the Viterbi algorithm captures the conditional dependencies between entities in the sequence. The outcome of the CRF layer provides the final tagging score for each entity.

**Fig 7:**
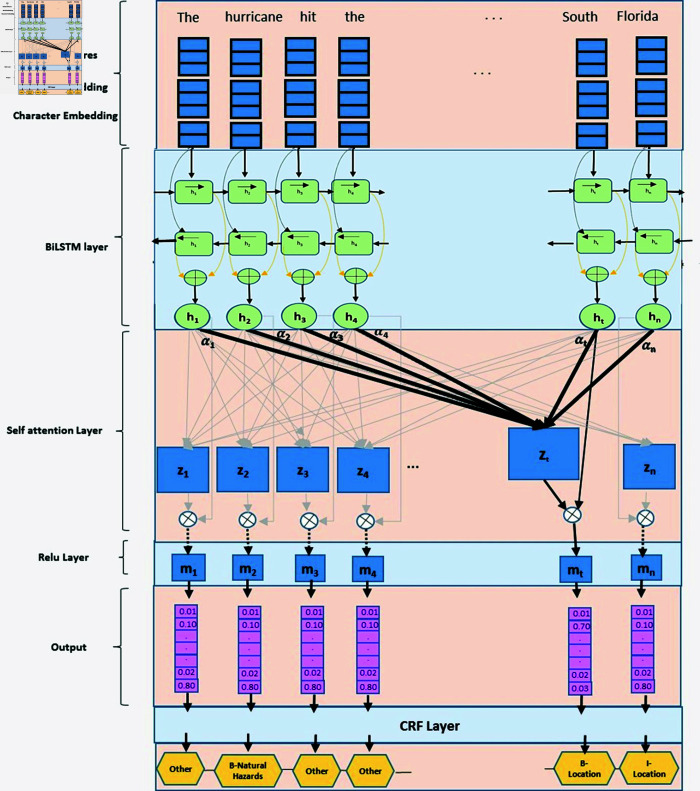
An illustration of the proposed BiLSTM-ATTN-CRF model architecture for disaster-related named entity recognition task.

In the following paragraphs, we will delve into the technical details of our Bi-LSTM-ATTN-CRF model, drawing insights from the work of Luo et al. [28]. By incorporating these techniques, our model aims to effectively address the sequence labeling task and achieve accurate classification of word tokens into their respective disaster classes. Fig 7 demonstrates an illustration of the proposed NER model.

Given an input sentence, $S=(w_{1},w_{1},w_{1},\ldots ,w_{n})$ that consists of n words, the task is to assign a tagging score to each word. The embedding and other NLP features including context-sensitive word embedding, character embedding, POS tagging and casing features for each word are provided as input to BiLSTM layer. The BiLSTM model predicts a label that corresponds to each of the word tokens in the sentence. Through using the embedding and other NLP features, the sentence is expressed as a sequence of vectors, $X=(x_{1},\ldots x_{i},\ldots ,x_{n})$, where *n* represents the number of words in the sentence or the length of the sequence. Given that input to BiLSTM layer, a forward and a backward LSTM network are used to compute two context representations of $\vec{h_{t}}\text{and}\overset{\leftarrow }{\left(h_{t}\right)}\text{of each word}t$, in the sentence from left to right and then in the reverse direction, respectively. The final representation of an individual word, $h_{t}=[\vec{h_{t}}:\overset{\leftarrow }{h_{t}}]$ is obtained by the concatenation of the forward and backward context representation of that word in the sentence. We used the Keras [29] Bidirectional LSTM model to implement the base BiLSTM layer. Following to that, a Self-Attention layer is added on the top of the BiLSTM layer to capture the contextual representation of each word by assessing its long-distance relationship with the rest of the words in the sentence. The attention weight value, $\alpha_{t,j}$ is calculated by computing the scaled dot-product between the “query” i.e. *x_t_*(*t^th^* current target word representation) and ‘*key*’ i.e. *x_j_*(*j^th^* word representation) in the sentence and then normalizing it by applying the ‘*Softmax*’ function.


$$\begin{array}{c} \alpha_{t,j}=softmax(x_{t}^{T}x_{j}) \end{array}$$ (1)

Following that, the Self-attention layer computes the context vector representation of the current target word, *z_t_*, by considering the attention-weighted version of the target word, *x_t_*, including all other words in the sentence as its context via the attention weights.


$$\begin{array}{c} z_{t}=\Sigma_{t=1}^{n}\alpha_{t,j}h_{t} \end{array}$$ (2)

We used Keras ‘*Attention*’ layer to implement the Self-Attention layer. Then a ‘*reLu*’ layer is used to predict the confidence scores for the word belonging to each of the possible labels as the output value of the BiLSTM-ATTN network.


$$\begin{array}{c} m_{t}=relu(W_{r}Z_{t}) \end{array}$$ (3)

Here, *W_r_* is the parameter of the model to be learned during the training. Finally, instead of modeling the tagging decisions context-independently, the CRF layer is added to the top of the Self-attention layer for contextual decoding of the best tag path for each target sequence. We assume that P is the matrix of output scores by the BiLSTM-ATTN network. Here, each element $P_{t,i}$ indicates the score of the $i^{t}h$ tag for the $t^{t}h$ current target word in the sentence. CRF will generate a tagging transition matrix, *T*, in which each element $T_{i,j}$ indicates the score of transition from tag *i* to tag *j*. The score of the input sentence *X* along with a tagging sequence $y=(y_{1},\ldots .,y_{t},\ldots .y_{n})$ is then calculated by combining the transition scores and network scores.


$$\begin{array}{c} s(X,y)=\Sigma_{t=1}^{n}T_{y_{t-1},y_{t}}+P_{t,y_{t}} \end{array}$$ (4)

The CRF model attempts to maximize the log probability score of the predicted tag sequence during the training phase using the Dynamic programming strategy employed in ‘*Viterbi*’. At the inference time, the model predicts the best tag path that obtains the maximum score. The ‘*CRF*’ from the Keras package is used to implement the CRF layer.

### BiLSTM-Attn-CRF modeling

The final BiLSTM-Attn-CRF model is achieved through the integration of the BiLSTM network, Multi-head Self-attention, and the CRF layer. This combination forms a fully differentiable neural network that is amenable to training using the conventional back-propagation technique. The modeling process encompasses several key steps, including the generation of training and test data, hyperparameter optimization, model development with optimized parameters, and ultimately, the evaluation of the model’s performance using independent test data. Each of these steps contributes to the comprehensive development and assessment of the final model.

#### Training and test data.

The NER model dataset consists of a total of 321086 disaster-related entries extracted from 1000 news articles (refer to Dataset generation). The news article dataset is then divided into training and test data with a ratio of 80%-20% split. The details of training and test data are presented below in Table 3. On the other hand, Table 4 shows the major disaster-related entity types in the news article dataset with their corresponding distributions in Training and Test data.

**Table 3:** Number of sentences, words, and characters in Training and Test data.

Set	Articles	Sentences	Words	Characters
**Train set**	800	399,636	80,062	2,374,391
**Test set**	200	41,766	83,446	262,002

**Table 4:** Distribution of major disaster-related named entities in training and test data.

Set	Train set	Test set
**Natural Hazards**	4668	618
**Location**	11564	1446
**Date**	7301	647
**Floods**	2144	230
**Fire**	918	29
**Death and toll**	1655	244
**Affected population**	1150	185
**Infrastructure damages**	588	248
**Collapsed structure**	345	82
**Water shortage**	67	30
**Power outage**	189	121

The tagging of the word tokens follows the abbreviated format of ‘B’ (Beginning), ‘I’ (Inside), and ‘O’ (Outside). When a single entity spans multiple tokens, the initial token is labeled with a ‘B’ prefix to indicate the beginning of the entity phrase, while the subsequent tokens bear an ‘I’ prefix to denote their position inside the entity. Conversely, non-relevant entities are assigned ‘O’ labels, representing the ‘Other’ entity type. Table 5 presents an illustrative example sequence that demonstrates word tokenization and the corresponding labeled entities.

**Table 5:** Example of tokenization and labeling in a sample sentence.

Token	Label
‘The’	O
‘Intensity’	O
‘Of’	O
‘The’	O
‘Drought’	B-NaturalHazards
‘In’	O
‘New’	B-Location
‘South’	I-Location
‘Wales’	I-Location
‘Has’	O
‘Left’	O
‘Soil’	O
‘Moisture’	O
‘Levels’	O
‘Severely’	O
‘Depleted’	O
‘.’	O

#### Hyper-parameter optimization.

Hyperparameters play a crucial role in determining the performance of deep learning models as they govern the learning process. Consequently, optimizing these hyperparameters becomes a vital step in building effective models, aiming to identify the ideal combination of hyperparameters that maximizes performance. In our study, we employed the grid search method, a widely-used approach for hyperparameter optimization. This method systematically explores various hyperparameter values to identify the optimal configuration. Specifically, we selected the following hyperparameters for optimization, “Learning rate", “Epoch", “Optimizer", “Hidden neurons", “Dropout", and “Activation Function". In order to fine-tune these six hyperparameters within a selected range of values, we created a development set by extracting 20% of the training dataset. The development set served as the basis for finding the best hyperparameter settings for the BiLSTM-ATTN-CRF model. During the hyperparameter tuning process, we utilized ‘Accuracy’ as the evaluation metric on the validation dataset. To determine the optimal epoch value, we employed an early stopping strategy. Interestingly, Stochastic Gradient Descent (SGD) did not emerge as the best optimizer for the current dataset. Instead, Root mean square propagation (rmsprop) exhibited superior performance. Among the three activation functions considered, ‘reLu’ demonstrated the best performance when applied to both the BiLSTM and dense layers. The final configuration of the BiLSTM-ATTN-CRF model was trained using the following hyperparameter values: number of epochs of 50, a learning_rate of 0.001, optimizer function as ‘rmsprop’, 100 hidden neurons in the BiLSTM layer, 150 hidden neurons in the dense layer, ‘reLu’ as the activation function, and a dropout rate of 0.2. Table 6 provides an overview of the range of values explored for each hyperparameter during the Grid search process, as well as the optimized values that yielded the best performance.

**Table 6:** The list of Hyper-parameters with corresponding ranges and optimal values.

Name	Range	Optimal value
Epochs	(20,30,50, 100)	50
Learning rate	(0.001, 0.005, 0.0001, 0.00001)	0.001
Optimizer	(NADAM, ADAM, rmsprop, SGD)	rmsprop
Hidden neurons	[(50,100),(100,100),(100,150),(200,256)]	(100, 150)
Activation function	(reLu, sigmoid, tanh)	reLu
Dropout	(0.2, 0.4, 0.6)	0.2

#### Model training.

The BiLSTM-ATTN-CRF model is trained using mini-batches of training sentences, employing the optimal hyperparameter values determined in the previous step. Each mini-batch comprises multiple sentences with a comparable number of tokens. This approach enables the model to learn from diverse examples and facilitates efficient parameter updates. The training procedure iterates over the mini-batches, performing updates for each batch of n training sentences. This strategy helps reduce the variance of parameter updates, promoting more stable convergence and enhancing the model’s learning capabilities. Throughout the training process, the model undergoes continuous evaluation of the development dataset. This evaluation aids in monitoring the model’s performance and facilitates early detection of potential issues or suboptimal learning. To address the challenge of overfitting, two techniques are employed. Firstly, dropout layers are incorporated into the model architecture. Dropout randomly deactivates a proportion of neurons during training, forcing the model to learn more robust and generalizable representations. This regularization technique helps prevent the model from relying too heavily on specific features or patterns in the training data, thus mitigating overfitting. Additionally, early stopping is applied during the training process. Early stopping involves monitoring the model’s performance on the development dataset and terminating the training if the performance no longer improves. This technique helps prevent the model from excessively fitting the training data, allowing it to generalize better to unseen examples. By combining dropout layers and early stopping, the model’s capacity to overfit is effectively controlled. These techniques enhance the model’s ability to generalize from the training data and improve its performance on unseen data, ultimately leading to a more robust and reliable BiLSTM-ATTN-CRF model for NER. It is pertinent to highlight that no effort was made to optimize the patience rate or minibatch size during model training. A patience rate of 3 (i.e. the number of epochs to continue if training loss stopped improving) was selected. As for the minibatch size, we tailored it to the sequence length for optimal performance. Consequently, each batch comprised sequences of identical lengths, thereby defining the respective minibatch size. The subsequent section delves into the optimization of the remaining model hyperparameters.

#### Model training time.

To ensure efficient and accelerated model training, we utilized the processing power of a Google Colab GPU. This specialized GPU (Graphics Processing Unit) is equipped with high-performance capabilities, enabling us to expedite the training process. As a result, the measured training time for the BiLSTM-ATTN-CRF model is found as approximately 2 hours. The utilization of a GPU significantly enhances the computational speed, allowing us to train models more swiftly and effectively.

#### Model evaluation metrics.

The performance of the model was assessed on the test data using four commonly employed evaluation metrics for NER in NLP. In the current study, we focused on Precision, Recall, Accuracy, and F1-score as the key metrics to measure the model’s effectiveness. It is worth noting that these metrics were evaluated at the sentence level, providing a more granular analysis rather than aggregating the results across the entire dataset. Precision, a metric that quantifies the proportion of correctly predicted named entities among all the predicted entities, was employed to assess the model’s ability to accurately identify relevant entities. Recall, on the other hand, measures the ratio of correctly predicted named entities to the total number of true entities, thereby capturing the model’s capacity to detect all relevant entities. Accuracy, as a general performance metric, represents the overall correctness of the model’s predictions compared to the ground truth labels. It offers a comprehensive measure of the model’s ability to correctly classify named entities. F1-score considers the harmonic mean of Precision and Recall, reflecting the model’s performance in achieving a trade-off between correctly predicting relevant entities (Precision) and not missing any relevant entities (Recall). By evaluating these four metrics at the sentence level, we gain insights into the model’s performance on a more localized basis. Particularly, a micro-average approach for these evaluation metrics is employed to address the dataset’s imbalance. The mathematical formulas for micro-average Precision (P), Recall (R), Accuracy (A), and F1-score (F1) are provided in Equations 6-9.


$$\begin{array}{ccc} & P=\frac{\sum TP}{\sum (TP+FP)} & \end{array}$$ (5)

$$\begin{array}{ccc} & R=\frac{\sum TP}{\sum (TP+FN)} & \end{array}$$ (6)

$$\begin{array}{ccc} & A=\frac{\sum (TP+TN)}{\sum (TP+FP+FN+TN)} & \end{array}$$ (7)

$$\begin{array}{cccc} & F1=\frac{2\times R\times P}{R+P} & & \text{(8)} \end{array}$$ (5)

In the above formulas, TP denotes the correctly classified disaster-related entities, whereas TN denotes the correctly predicted non-disaster entities. Further, FP indicates the number of predicted disaster entities that are non-disaster entities, and FN indicates the number of disaster entities wrongly predicted as non-disaster entities.

## Results and discussion

### NER performance comparison for different model configurations

The BiLSTM-ATTN-CRF model consists of three major components: a BiLSTM layer, a Self-Attention layer, and a CRF layer. These layers incrementally add complexity to the model. Multiple experiments were conducted to assess the model’s accuracy as the complexity increased beyond the baseline BiLSTM network. Additionally, two different model configurations, namely the BiLSTM-Attention model and the BiLSTM-CRF model, were tested in these experiments. A total of four different model configurations were trained and evaluated using an independent test set, with the results presented in Table 7. The objective of these experiments was to examine how the model’s accuracy varied with increasing complexity. Interestingly, only the baseline BiLSTM model achieved good performance, with 88% precision, 91% recall, 84% accuracy, and 91% F1-score. The addition of the self-Attention layer improved the performance compared to the baseline BiLSTM model. On the other hand, the BiLSTM-CRF model demonstrated comparable performance to the BiLSTM-Attention architecture. Notably, the combined model, i.e., BiLSTM-ATTN-CRF, delivered the best performance across all four evaluation metrics. Specifically, precision and accuracy increased by 10% and 7%, respectively, compared to the second-best performing model. The BiLSTM-ATTN-CRF model and similar combinations have been successfully utilized in various NER-related tasks, leveraging contextual information and mitigating label inconsistency issues [27,28].

**Table 7:** Results on test data for different model configurations.

Method	Precision	Recall	Accuracy	F1-score
BiLSTM	0.889 ± 0.14E-2	0.917 ± 0.62E-2	0.846 ± 0.35E-2	0.903 ± 0.91E-2
BiLSTM-ATTN	0.911 ± 0.77E-2	0.913 ± 0.38E-2	0.856 ± 0.69E-2	0.912 ± 0.58E-2
BiLSTM-CRF	0.915 ± 0.45E-2	0.906 ± 0.19E-2	0.861 ± 0.72E-2	0.911 ± 0.36E-2
**BiLSTM-ATTN-CRF**	**0.925 ± 0.68E-2**	**0.911 ± 0.83E-2**	**0.868 ± 0.44E-2**	**0.918 ± 0.17E-2**

A comprehensive investigation reveals that when integrated atop a BiLSTM network, the self-Attention layer effectively captures the interaction between past and future context within each time interval. The token-level self-attention mechanism employs query, key, and value vectors to project hidden states into different feature subspaces, enabling consultations with other hidden states, similarity checks with incoming tokens, and information transfer to the input token. Furthermore, with multiple attention heads operating in parallel, the self-Attention model can focus on different aspects of the NER task [30]. Conversely, the CRF layer proficiently models the conditional dependencies among the output labels generated by the BiLSTM-ATTN, using a matrix that assigns transition scores between all pairs of labels. By effectively learning the internal syntax logic between labels, the CRF layer correctly annotates that I-LOC must follow B-LOC, while vice versa is not possible. This capability enhances the model’s ability to capture label dependencies and facilitate accurate annotation.

### The effect of contextual embedding for NER performance

The effectiveness of word embedding features extracted from contextual word databases surpasses that of non-contextual vector representations for query word tokens. To support this hypothesis, a comparative analysis was conducted using both contextual and general word embeddings. The general word representation refers to non-contextual table look-up for determining the disaster entity associated with the query word tokens. For generating non-contextual word representations, we utilized Word2Vec [15] and Glove [31] tools. On the other hand, our context-sensitive word embedding model produced the contextual word representation. Table 8 displays the performance of the BiLSTM-ATTN-CRF model using three different word embedding features. Contextual embedding features exhibited significantly superior performances compared to Word2Vec and GloVe features, achieving 92% precision, 91% recall, 87% accuracy, and 92% F1-score. Among the two general embedding models, GloVe embedding demonstrated relatively better performance (90% vs. 89%) than Word2Vec in recognizing disaster-related named entities. Previously, the GloVe embedding model showed competitive performances in the NER task by relying on global word-word co-occurrence features within a weighted least square architecture [32,33]. As the GloVe model’s training database was constructed from a large corpus of frequent words extracted from Wikipedia and other websites, it effectively recognized entities such as Location and Date. GloVe embedding features also showed moderate accuracy in extracting common entities within the ‘NaturalHazards’ class, such as hurricanes, rains, tornadoes, etc. Conversely, Word2Vec exhibited relatively poorer performance in extracting Location and Date entities but demonstrated competitive performance in recognizing terms within the ‘Floods’ and ‘InfrastructureDamage’ classes. However, when using contextual embedding as input features, the model excelled in extracting the majority of disaster-related entities with high precision, recall, and accuracy (>90%). This was observed in entities belonging to ‘Floods’, ‘NaturalHazards’, ‘InfrastructureDamage’, ‘Death_and_toll’, ‘CollapsedStructure’, and others. It is worth noting that contextual embedding did not perform well in cases where named entities had a low number of training or test samples, such as ‘RoadBlocked’, ‘WaterShortage’, and other class entities. Overall, the results indicate the clear superiority of contextual embedding features over non-contextual representations, with contextual embeddings significantly enhancing the model’s ability to accurately extract disaster-related entities across various classes. This analysis adds novel insights to the field and enhances our understanding of the effectiveness of contextual word embeddings in disaster management scenarios.

**Table 8:** BiLSTM-ATTN-CRF model performances using different word-level embeddings.

Embedding feature	Precision	Recall	Accuracy	F1-score
Word2vec	0.854 ± 0.57E-2	0.930 ± 0.40E-2	0.822 ± 0.83E-2	0.890 ± 0.65E-2
Glove	0.916 ± 0.22E-2	0.900 ± 0.69E-2	0.849 ± 0.13E-2	0.908 ± 0.29E-2
**Contextual**	**0.925 ± 0.68E-2**	**0.911 ± 0.83E-2**	**0.868 ± 0.44E-2**	**0.918 ± 0.17E-2**

### NER performance comparison for different feature combinations

We investigated the performance of the BiLSTM-ATTN-CRF model by exploring different combinations of features in various layers. Initially, we augmented the baseline embedding features (word and character-level embeddings) with additional features, such as POS tagging and casing information. These combined features were fed into both the BiLSTM layer and the Self-Attention layer. Additionally, we explored three more feature combinations. In the first combination, the additional features were concatenated with word and character embeddings and fed into the BiLSTM layer, while the Self-Attention layer computed weight values using only the baseline embedding features. For the second combination, the word and character level embeddings were input solely to the BiLSTM layer, while the additional features, along with word and character embeddings, were utilized to compute the Self-Attention weight values. In the final experiment, the baseline embeddings were exclusively input to both BiLSTM and Self-Attention layers without integrating any supplementary NLP features. These four feature combinations resulted in four distinct entity classification models. We evaluated these models using an independent test dataset, and the results are presented in Table 9. The table showcases the four evaluation metrics and demonstrates that the four feature combinations yield comparable performances. Significantly, the feature combination encompassing all features consistently exhibited superior values across all evaluation metrics, contrasting with the exclusive use of baseline embeddings, which exhibited the lowest performance in the assessments. This outcome underscores that the BiLSTM-ATTN-CRF model performs optimally when the additional NLP features are combined with baseline embedding features and provided to both the BiLSTM and Self-Attention layers, signifying the contribution of POS tagging and Casing features in the NER classification. Integrating these diverse features enhances the model’s ability to capture relevant information and contribute to improved performance in entity classification tasks.

**Table 9:** BiLSTM-ATTN-CRF performances with various feature combinations. In column 1, “All" indicates the combinations of baseline embeddings, POS Tagging, and casing features, whereas “Base embeddings" indicates the word and character embedding features combined.

Features (Input layer(s))	Precision	Recall	Accuracy	F1-score
**All (BiLSTM+Self-Attn)**	**0.925 ± 0.68E-2**	**0.911 ± 0.83E-2**	**0.868 ± 0.44E-2**	**0.918 ± 0.17E-2**
All (BiLSTM), Base embeddings (Self-Attn)	0.911 ± 0.21E-2	0.898 ± 0.87E-2	0.849 ± 0.72E-2	0.905 ± 0.93E-2
Base embeddings (Bi-LSTM), All (Self-Attn)	0.903 ± 0.75E-2	0.913 ± 0.32E-2	0.853 ± 0.55E-2	0.908 ± 0.70E-2
Base embeddings (Bi-LSTM +Self-Attn)	0.837 ± 0.46E-2	0.770 ± 0.15E-2	0.754 ± 0.07E-2	0.802 ± 0.86E-2

### NER performance comparison with existing models and dataset

In the existing literature, there are only a few studies that specifically address the problem of disaster-related named entity recognition (NER) using either structured or semi-structured data. Recently, a study focused on a Mandarin language dataset and employed a transfer learning approach to tackle the disaster NER problem [34]. The authors aimed to identify six named entities, such as location, product_name, person_name, time, and org_name, which are relevant to disaster management. They reported model training evaluation results with F1-scores ranging from 51% to 94%. Another study by Cruz et al. [7] explored the extraction of disaster-related named entities from a dataset of Filipino news articles, utilizing deep learning techniques. The entities investigated included type, name, month, location, and others. The authors achieved the highest F-measure of 76% when evaluating their LSTM+CRF model on the test set. In a more recent study, Eligüzel et al. [35] employed RNN-based deep learning models to successfully extract named entities such as organizations, persons, and locations from earthquake-related tweets. Similarly, Sufi et al. [36] introduced an AI-based decision support system that utilized social media feeds to identify and analyze natural disaster events, aiming to generate intelligent insights for effective disaster management and strategic planning. Conversely, Sun et al. [37] explored multiple hybrid deep learning approaches to tackle the natural hazard named entity recognition task across three distinct categories: geographical locations, natural hazards, and research methods. The researchers achieved remarkable results, reporting an impressive Precision score of 92.8%, Recall of 91.74%, and an *F*_1_-score of 92.27% using the XLNet-BiLSTM-CRF model for this three-class natural hazard NER task. In contrast to recent studies, our current research takes advantage of contextual word embeddings and various NLP features integrated into a BiLSTM-ATTN-CRF model architecture. This approach enables the identification of 14 distinct named entities related to disasters, including classes such as NaturalHazards, Location, Date, Death_and_Toll, InfrastructureDamage, and CollapsedStructure, among others. Impressively, our model achieves outstanding results, with an overall Precision of 92.5%, Recall of 91.4%, Accuracy of 86.8%, and an *F*_1_-score of 91.8% when extracting terms from these 14 disaster-related ontology classes. These results are summarized in Table 10. Based on the findings from these pertinent studies, we conducted a systematic experiment in which we trained alternative models using our datasets. Subsequently, we evaluated the performance of our model on publicly available NER datasets.

**Table 10:** Summary of performances in recent and current disaster-specific NER study.

Author (Year)	Disaster-related Entities	Method	F1-score
Cruz et al. (2018)	Type, month, location, and others	Deep Learning	76%
Kung et al. (2020)	Location, product_name, person_name, time, and org_name	Deep Transfer Learning	51-94%
Eligüzel et al. (2022)	Organizations, Persons, and Locations from earthquake-related tweets	AI-based decision support system	90%
Sufi et al. (2022)	Natural disaster events	RNN-based Deep Learning	92%
Sun et al. (2022)	Geographical locations, natural hazards, and research methods	XLNet-BiLSTM-CRF	92%
**Current Study (2024)**	**14 disaster-specific entities including NaturalHazards, Location, Date, Death and Toll, InfrastructureDamage, and CollapsedStructure, among others**	**Contextual BiLSTM-ATTN-CRF**	**92**%

#### Alternative approaches.

Among different techniques of NER modeling, the auto-encoder and decoder-based Bi-LSTM and bi-directional transformer-based models are prominent. In our study, we adopted the Bi-LSTM architecture augmented by additional layers of Self-attention and CRF. However, it is worth investigating the potentiality of pre-trained language models for the present NER problem. Hence, in addition to exploring various configurations of the Bi-LSTM model, we investigated the effectiveness of the Biconditional Encoder Representations from Transformers (BERT) model [38] in classifying disaster-related named entities using the concept of transfer learning. Specifically, we chose BertForTokenClassification [39], which incorporates BERT as its base architecture, accompanying a token classification head on top. Rather than at the sequence level, this model makes predictions at the token level, making it suitable for the current NER task. We utilized the pre-trained weights of “bert-base-uncased" to initialize the base layers of the model. The top layer, a token classification head with randomly initialized weight, was trained using our labeled disaster-specific training dataset. The fine-tuning of the pre-trained BERT model was performed using ‘ADAM’ optimizer and a learning rate of 10^−5^. Furthermore, a BERT-based contextual NER model was implemented. In this experiment, BERT’s contextual embeddings were generated utilizing our training dataset. Subsequently, these embeddings were fed into the BiLSTM layer, followed by dense and classification layers with ‘softmax’ as an activation function. The primary goal was to assess the impact of BERT’s contextual embeddings on the NER task. Finally, we evaluated these models’ performance on the holdout test set. The results of the sentence-level evaluation, as shown in Table 11, revealed that the fine-tuned BERT model achieved slightly lower performance compared to the baseline BiLSTM model, with precision of 88%, recall of 78%, accuracy of 80%, and F1-score of 83%. This outcome can be attributed to two factors. Firstly, BERT is primarily pre-trained on diverse NLP tasks such as Masked Language Modeling and Next Sequence Prediction, which may not fully align with our specific domain of disaster-related named entity classification. Secondly, fine-tuning BERT with a simple supervised training approach, without incorporating meaningful feature extraction techniques, may limit its capacity for optimal learning and performance improvement. Conversely, the BERT’s contextual embedding-based classification exhibited precision of 91%, recall of 86%, accuracy of 82%, and an F1-score of 88%. These results signify an enhancement in the performance of the NER through the incorporation of BERT’s contextual embedding during the classification process.

However, by incorporating our custom contextual word embeddings and employing an augmented BiLSTM model architecture, our study represents significant progress in disaster NER, surpassing the performance of the pre-trained language models.

**Table 11:** NER performance comparative analysis for alternative model configurations.

Model	Precision	Recall	Accuracy	F1-score
Fine-tuned BERT	0.888 ± 0.59E-2	0.786 ± 0.25E-2	0.803 ± 0.37E-2	0.834 ± 0.61E-2
Contexual BERT	0.910 ± 0.34E-2	0.861 ± 0.71E-2	0.821 ± 0.70E-2	0.884 ± 0.16E-2
**Our model**	**0.925 ± 0.68E-2**	**0.911 ± 0.83E-2**	**0.868 ± 0.44E-2**	**0.918 ± 0.17E-2**

**Table 12:** NER performance comparative analysis for alternative dataset.

Entity Class	Date			Location			Others		
Dataset	Prec	Rec	F1	Prec	Rec	F1	Prec	Rec	F1
**GMB**	0.915	0.38	0.48	0.68	0.4	0.50	0.97	0.98	0.97
**Our Dataset**	0.97	0.97	0.97	0.86	0.885	0.87	0.99	0.99	0.99

#### Alternative dataset.

It is worth noting that we encountered a significant gap in the existing literature, as there is currently no publicly available annotated dataset specifically focused on disaster-related English news articles. Given these limitations, we conducted a comprehensive generalizability test of our NER model using the Groningen Meaning Bank (GMB) corpus NER [40]. The GMB corpus, a publicly available dataset comprising multi-sentence English texts accompanied by lexical and semantic representations, has been extensively employed in diverse studies for evaluating NER models [41–43]. This corpus encompasses commonly encountered entities, including ‘Date’ and ‘Location’. For our model evaluation, the non-disaster-related entities in the GMB corpus were re-labeled as ‘Other’. To assess the performance of our NER model in classifying three distinct entity types (‘Date’, ‘Location’, and ‘Other’), we chose a subset of the GMB dataset consisting of initial 10,000 entries. The evaluation results are presented in Table 12. The results indicate that our model achieved precision rates of 91% for ‘Date’, 68% for ‘Location’, and 97% for ‘Other’ entity classes, with corresponding recall rates of 38% for ‘Date’, 40% for ‘Location’, and 98% for ‘Other’. These figures underscore the effectiveness of our NER model within this specific subset of the GMB corpus. The subsequent row illustrates the model’s performance for the respective entities in the current dataset. A close inspection reveals that our model encountered challenges in accurately identifying certain ‘Date’ and ‘Location’ entities within the GMB dataset due to formatting discrepancies. Nevertheless, the relatively higher precision values affirm the model’s ability to distinguish positive entities likely to be correct. The assessed generalization performance underscores our proposed methodology’s effectiveness and potential impact.

## Challenges and future research

The current research faced a significant challenge in annotating entities with appropriate disaster-related ontology classes. The presence of overlapping class definitions from multiple ontologies and the potential for entities to have multiple class memberships made the annotation process exceptionally complex. To address this challenge, the researchers sought guidance from domain experts in the field of disasters, which proved invaluable. Moreover, certain disaster ontology classes had limited entries due to their infrequent occurrence in the dataset of disaster articles. In light of these challenges and findings, several potential directions for future research can be explored in the current study. Firstly, expanding the news articles dataset by annotating a larger number of articles would enhance its coverage and depth. Secondly, to ensure the reliability of the annotation process, further strategies regarding designing a clear annotation guideline, building an expert annotation team, promoting annotation consistency by exercising inter-annotator agreements, and introducing an iterative annotation review process could be adopted. Thirdly, incorporating more frequently occurring disaster-related ontology classes into the annotated dataset would provide a richer representation of the domain. Lastly, exploring more advanced architectures capable of extracting contextual information from named entities could further improve the accuracy and effectiveness of the NER task. By addressing these potential avenues for future research, the current study can continue to advance the field of disaster-related entity recognition and contribute to the development of more robust and comprehensive disaster ontologies.

## Conclusion

In this study, we have curated a unique dataset comprising 1000 news articles focused on disaster-related topics. To enhance the dataset’s value, we have annotated it with 14 distinct types of disaster entities, utilizing vocabularies derived from the MOAC [13] and Empathi [14] disaster ontologies. Our investigation into identifying these disaster-related named entities involved a context-aware self-attentive NLP model called BiLSTM-ATTN-CRF. To further improve the model’s performance, we have developed a context-specific word embedding model and an enhanced character embedding model. These models enable us to generate customized word and character-level embedded features. Through a comparative analysis, we have determined that the use of context-specific word representations significantly enhances the recognition of disaster-related named entities. Specifically, our BiLSTM-ATTN-CRF model, incorporating context-specific word embedding, outperforms models utilizing word2vec and glove-embedded features, and fine-tuned language models like BERT. It achieves a Precision of 92.5%, Recall of 91.4%, Accuracy of 86.8%, and an F1-score of 91.8%. This research demonstrates the effectiveness of integrating context-specific word embedding in accurately identifying disaster-specific named entities within a novel dataset of news articles focused on disasters. The findings highlight the utility and potential impact of our approach in the field of disaster-related entity recognition.
